# The emerging function and clinical significance of circRNAs in Thyroid Cancer and Autoimmune Thyroid Diseases

**DOI:** 10.7150/ijbs.55381

**Published:** 2021-04-17

**Authors:** Yu Zhang, Dong-Dong Jia, Yi-Fei Zhang, Meng-Die Cheng, Wen-Xiu Zhu, Pei-Feng Li, Yin-Feng Zhang

**Affiliations:** 1 Institute for Translational Medicine, The Affiliated Hospital of Qingdao University, College of Medicine, Qingdao University, Deng Zhou Road 38, Qingdao 266021, China.; 2 Department of Cardiology, The Affiliated Hospital of Qingdao University, Qingdao, China. Institute for Translational Medicine, Qingdao University, Qingdao, China.

**Keywords:** circular RNA, thyroid cancer, autoimmune thyroid diseases, biomarker, therapeutic target

## Abstract

Thyroid cancer (TC) is one of the most common malignant tumors, with high morbidity and mortality rates worldwide. The incidence of TC, especially that of papillary thyroid carcinoma (PTC); has increased rapidly in recent decades. Autoimmune thyroid disease (AITD) is closely related to TC and has an estimated prevalence of 5%. Thus, it is becoming increasingly important to identify potential diagnostic biomarkers and therapeutic targets for TC and AITD. Circular RNAs (circRNAs) are a class of non-coding RNAs with covalently bonded circular structures that lack 5'-3' polarity and polyadenylated tails. Several circRNAs play crucial roles in the development of various diseases, including TC and AITD, and could be important new biomarkers and/or targets for the diagnosis and therapy of such disorders. Although there are four subtypes of TC, research on circRNA has largely focused on its connection to PTC. Therefore, this review mainly summarizes the relationships between circRNAs and PTC and AITD, including the molecular mechanisms underlying these relationships. In particular, the functions of “miRNA sponges” and their interactions with proteins and RNA are discussed. The possible targeting of circRNAs for the prevention, diagnosis, and treatment of TC and AITD is also described. CircRNAs could be potential biomarkers of TC and AITD, although validation will be required before they can be implemented in clinical practice.

## Introduction

### 1.1 Thyroid cancer and autoimmune thyroid diseases

Thyroid cancer (TC) has long been considered to arise in middle age and, after repeated proliferation, resulting in further damage to the genome, can progress to more aggressive and lethal types of cancer 1. Three major studies published in 2014 led to a significant change in our understanding of the natural history of TC 1-5. These studies, known as the 2014 TC trilogy, were the first to report the existence of self-limiting cancers 6-10. There are four subtypes of TC, namely papillary thyroid carcinoma (PTC), follicular thyroid carcinoma, medullary thyroid carcinoma, and anaplastic thyroid carcinoma. PTC accounts for about 80% of TC followed by follicular carcinoma, which accounts for about 10% 11. Autoimmune thyroid diseases (AITDs), which are related to TC 12, are caused by a dysregulation of the immune system that results in an immune attack on the thyroid. AITDs such as chronic lymphocytic thyroiditis and Graves' disease (GD) are B cell- and T cell-mediated organ-specific autoimmune disorders 13, 14. Chronic lymphocytic thyroiditis was first proposed by the Japanese scholar Hashimoto in 1912, hence it is also known as Hashimoto's thyroiditis (HT) 15.

Although TC was once considered a relatively uncommon malignancy, in general, the incidence of TC has been increasing year by year due to various potential factors including environmental contaminations 16, 17. Indeed, long-term, high-quality registry data have revealed that the incidence of the disease has increased substantially over the past few decades in almost every country 11, 18. Approximately 567,000 cases of TC are reported worldwide, and the disease was ranked in ninth place for worldwide cancer incidence in 2018 19. Throughout the world, the incidence of TC is approximately three times higher in women (6.1 per 100,000 per year) than in men (1.9 per 100,000 per year) 20. Initially, palpation was the predominant means of finding thyroid nodules, and surgery was necessary to diagnose TC 21. Later, technical improvements (including the development of new forms of tracers and pin-hole collimation) enabled thyroid scans to become more sensitive than palpation 22. The most recent advance in thyroid imaging is the use of high-definition ultrasonography; however, although this technique is highly sensitive, it lacks specificity because most thyroid nodules are benign, and very small cancers may not be clinically significant 21. Currently, sampling via fine-needle aspiration (FNA) is used to guide the therapeutic management of patients with thyroid nodules. Indeed, the use of FNA can reduce unnecessary thyroid surgery by 25% 23. Nonetheless, the prevalence of FNA samples with indeterminate and non-diagnostic cytology remains high at approximately 30% 23. Furthermore, because the prognosis of most cases of TC is so good that controlled studies are almost impossible, there are still many unanswered questions regarding the best form of treatment 21. Consequently, there is a need to focus more efforts on the effective diagnosis, control, and treatment of TC. In particular, understanding the underlying molecular mechanisms of TC will aid the development of effective individualized treatments.

AITD has an estimated prevalence of 5%, making it one of the most widespread autoimmune diseases 14. The criteria used for diagnosis of AITD include circulating concentrations of autoantibodies and thyroid stimulating hormone, as well as clinical and biochemical features, such as classical features on thyroid ultrasounds. By contrast, FNA cytology and radioactive iodine absorption are rarely used as diagnostic indicators of AITD 12. Treatment of the hypothyroidism that arises as a result of AITD includes daily consumption of synthetic L-T4 (1.5-1.7 µg/kg body weight/day) 12. However, there remain major challenges in providing effective healthcare for AITD patients, such as accurate diagnosis, choosing the right treatment, accurately predicting therapeutic responses, and providing appropriate personalized treatments 24. Thus, it is important to explore new potential biomarkers and therapeutic targets of AITD.

### 1.2 Circular RNAs

#### 1.2.1 Structure, classification, and biogenesis of circRNAs

**Figure 1:**
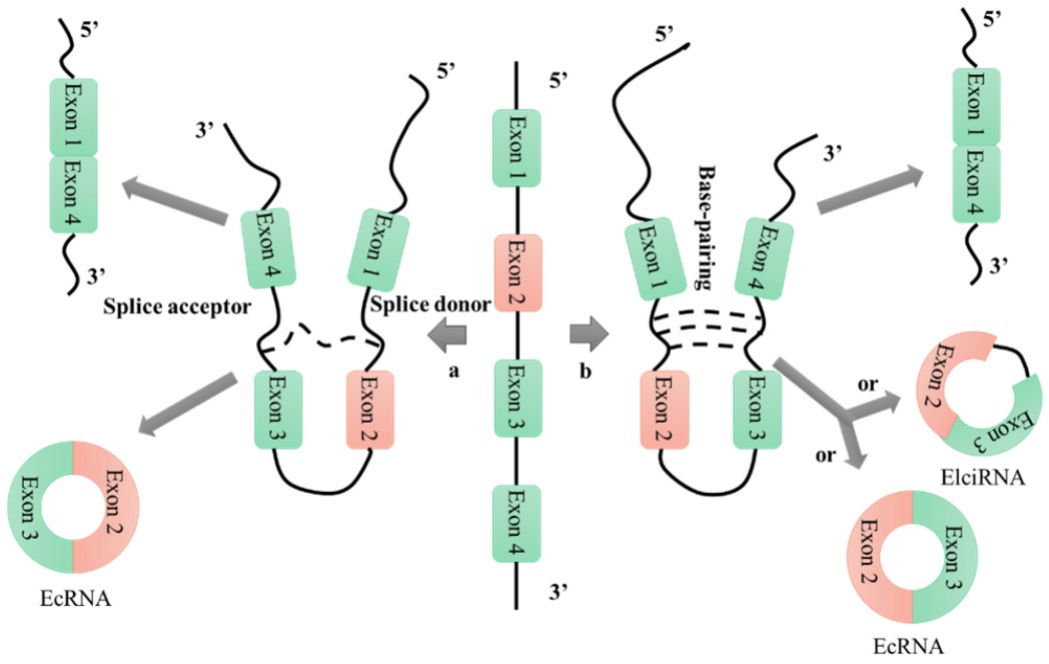
CircRNA formation mechanisms. a: lariat-driven circularization; b: intron-pairing-driven circularization.

Based on their patterns of biogenesis from genomic regions, circular RNAs (circRNAs) can be divided into four categories: exonic circRNAs, circular intronic RNAs, exon-intron circRNAs, and intergenic circRNAs 25, 26. There are many hypotheses for the mechanism of circRNA formation, among which the lariat-driven circularization and intron-pairing-driven circularization mechanisms are the most widely accepted (**Figure 1**) 27. Research into the formation of circRNAs has had a positive catalytic effect on the exploration of their functions; however, many proposed mechanisms require further analysis and verification. Unlike linear RNAs, which are terminated with 5' caps and 3' tails, circRNAs are characterized by covalently closed loop structures that lack 5' to 3' polarity and polyadenylated tails. This intrinsic characteristic has led to a general underestimation of the existence of circular RNAs in previous polyadenylated transcriptome analyses 28. However, in recent years, RNA sequencing technologies and bioinformatics approaches have identified a large number of circRNAs in humans 29-31, mice 29, 30, nematodes 29, 32, zebrafish 33, fruit flies 34, protists 35, and plants 35. Furthermore, these technologies have enabled characterization of the abundance and diversity of circRNAs, as well as their dynamic expression patterns in various developmental stages and physiological conditions.

Many circRNAs are expressed in a tissue-specific manner. For example, circRmst and circKlhl2 are expressed at high levels in the mouse brain but are not found in the liver or lung 36, and circ-MBL expression in the Drosophila ovary is much lower than that in the head 37. The expression levels of some circRNAs also change at different ages or developmental stages. For example, one study found that the levels of multiple circRNAs, including MM9_circ_004501 and MM9_circ_013636, were higher in the brains of 22-month-old rats than in those of 1-year-old rats 29.

#### 1.2.2 Functions of circRNAs

To date, circRNAs have been reported to function as competing endogenous RNAs or “miRNA sponges” 26, 38, 39, 40, mediators of RNA/protein interactions 39, and scaffolds in the assembly of protein complexes 42. In addition, circRNAs can be translated into functional proteins, sequester proteins from their native subcellular localization 43, modulate the expression of parental genes 38, 44-46, regulate alternative splicing 41, and modulate the stability of mRNAs 26.

The ability of circRNAs to function as miRNA sponges has been studied in most detail. The presence of miRNA binding sites or response elements in some circRNAs enables them to regulate the function of miRNAs by acting as competitive endogenous RNAs (ceRNAs) or “miRNA sponges” 26, 40, 46, 47. Competitive binding of miRNAs to ceRNAs reduces the expression levels of the free miRNAs and inhibits their ability to affect gene expression at the transcriptional or post-transcriptional level 25, 48, 49. However, the biological significance of the classic role of ceRNAs as miRNA sponges is being increasingly debated, and a recent study demonstrated that most circRNAs do not function in the same way as “real” miRNA sponges 50.

The protein interactions of a number of circRNAs, such as circ-Foxo3 39, 42 and circ008274 51, have been studied extensively in disease states, especially tumors. Ectopic expression of circ-Foxo3 inhibits cell cycle progression by binding to the cell cycle proteins cyclin-dependent kinase 2 and cyclin-dependent kinase inhibitor 1 (p21) 39.

Moreover, a number of endogenous circRNAs can be translated into proteins 52-54, which calls into doubt the notion that they function as non-coding RNAs exclusively. Other studies have shown that synthesized circRNAs containing multiple FLAG-coding sequences can also be translated into proteins in the absence of a particular element for internal ribosome entry, through a mechanism similar to rolling circle amplification 55.

CircRNAs can also regulate gene transcription. For example, circ-EIF3J and circ-PAIP2 can combine with the U1 small nuclear ribonucleoprotein to interact with RNA polymerase II and enhance the expression of their parental genes in HeLa and HEK2393 cells 44.

The enormous number of circRNAs identified to date suggests that they may have complex and diverse functions. In terms of characterizing the mechanisms underlying tumor formation and development, the roles of circRNAs as miRNA sponges have been studied in most detail, followed by their interactions with proteins. However, the relationships between cancer and the abilities of circRNAs to be translated into proteins, modulate the stability of mRNAs and the splicing of pre-RNAs, and regulate parental gene transcription have not been studied in detail. These potential new roles of circRNAs in tumor development require further investigation.

#### 1.2.3 CircRNAs as tumor markers

Recently, genome-wide analyses have shown that various circRNAs play a role in a number of pathological processes, including the development of cancer, through transcriptional and post-transcriptional regulatory mechanisms 56-58. The identification of differences in gene expression levels between tumor and normal samples is an important component of cancer biology research. Analyses of a variety of tumor cells and corresponding normal cells have revealed abnormal circRNA expression levels in a variety of cancers 58. In addition, other studies have shown that circRNAs participate in the initiation and progression of tumors, and can be used as molecular markers of the activation of signal transduction pathways related to cancer development 59-63.

Although a large number of studies have examined the use of proteins and miRNAs as biomarkers of cancer 64, 65, circRNAs have a number of unique advantages. The main advantages of circRNAs as cancer biomarkers include their ability to be detected via RT-PCR analyses of minimally invasive blood, urine, or saliva samples 43, 66, 67, as well as their high circulating stability. Indeed, circRNAs are unusually stable RNA molecules, presumably because they lack an open end and are therefore able to avoid conventional RNA degradation pathways 58. Notably, circRNAs play an important role in regulating the Wnt/β-catenin pathway 43, 68, 69, an important signaling mechanism involved in tumor progression 70. It is possible that targeted inhibition of circRNAs could block the Wnt/β-catenin pathway to inhibit tumor growth and development.

## 2. CircRNAs and TC

In recent years, several studies have shown that a number of circRNAs are abnormally expressed in tumor tissues and are related to cancer progression 71. Differential expression of these circRNAs can lead to changes in both biological processes and the flow of genetic information. CircRNAs play roles in TC signaling pathways and the invasion and migration of TC cells. In addition, the expression levels of circRNAs can be used for diagnosis of TC and monitoring the response to therapy. **Table 1** summarizes the changes in expression levels (i.e., up-regulation or down-regulation) and regulatory functions of circRNAs in TC reported in recent studies. However, due to the high proportion of PTC among all thyroid cancers (about 80%) 11, recent studies on circRNA and TC have largely focused on PTC.

The link between inflammation and cancer is well known. Over the past few decades, many studies have shown that AITD and TC (mainly PTC) can occur simultaneously, and that the increase in TC incidence coincides with the increase in AITD registration. However, the mechanism responsible for the link between AITD and TC is still unclear 72. Therefore, in this review, TC and AITD are described together in the hope that future studies will uncover the link between these two diseases and their relationships to circRNA levels.

### 2.1 Expression levels of circRNAs in TC

Recent studies have demonstrated that a number of circRNAs are expressed at abnormal levels in TC 71, including PTC. A microarray analysis of six PTC tumors, six matched normal control samples, and 18 thyroid samples from six benign thyroid lesions revealed that circRNA imbalance may play a role in the pathogenesis of PTC, and identified some key circRNAs as candidate biomarkers of the disease 73. Another study using a high-throughput sequencing analysis found altered levels of exosomal circRNAs in the serum of patients with PTC 74. Specifically, compared with those from the control group (patients diagnosed with benign goiter), serum-derived exosomes from PTC patients contained three up-regulated circRNAs and 19 down-regulated circRNAs. These differentially expressed circRNAs were related to 16 signaling pathways 74. Abnormal expression of circRNAs in PTC may result in changes in tissue function and metabolism, thereby representing a molecular mechanism underlying the development of PTC.

### 2.2 The emerging roles of circRNAs in TC proliferation, invasion, and progression

Abnormal cell proliferation, migration, and invasion are the main characteristics of TC. Studies performed in recent years have shown that circRNAs may be associated with these abnormal characteristics of TC cells.

Indeed, several circRNAs are closely associated with TC cell proliferation. Most of the circRNAs that promote thyroid cell proliferation exert their roles by functioning as miRNA sponges 75-79. The expression level of circZFR is correlated with the clinical severity of PTC 75. CircZFR reportedly promotes the expression of C8orf4 by acting as a ceRNA that targets miR-1261, thereby enhancing the proliferation, migration, and invasion of PTC cells. Moreover, Ye et al. showed recently that circFOXM1 promotes the progression of PTC by modulating the miR-1179/HMGB1 pathway 77. Another study found that circ-LDLR regulates LIPH expression by acting as a miRNA sponge targeting miR-195-5p, thereby promoting PTC progression 78. Li et al. showed that circNUP214 may play an oncogenic role in PTC by acting as a sponge for miR-145, leading to up-regulation of its target zinc finger E-box binding homeobox 2 79. In addition, Ma et al. reported that direct targeting of miR-1233-3p by circTP53 increases the level of the MDM2 mRNA and reduces p53 expression in TC cells, thereby promoting cell proliferation 76. In addition to these circRNAs that act as miRNA sponges, Bi et al. found that circ-102171 functions as an oncogene in PTC by interacting directly with the CTNNBIP1 protein. This interaction promotes cancer development by activating the β-catenin pathway 70.

In addition to the stimulatory effects of specific circRNAs on TC cell proliferation and migration, several circRNAs promote the metastasis or lymph node metastasis of these cells. Xue's group demonstrated that the level of circPRMT5 is positively correlated with advanced stage PTC and lymph node metastasis 80. Further investigation revealed that circPRMT5 functions as a competing endogenous RNA for miR-30c. The inhibition of miR-30c by circPRMT5 promotes the expression of E2F3, a crucial oncogene in several tumors. Sun et al. demonstrated that circ_0058124 might play a pro-metastatic role in TC by regulating the miR-940/MAPK1 axis 81. In addition, Yao et al. found that circ_0058124 acts as an oncogenic driver that promotes PTC cell proliferation, tumorigenicity, tumor invasion, and metastasis. Specifically, circ_0058124 functions as a competing endogenous RNA to modulate the expression levels of miRNA-218-5p and its target gene NUMB, leading to repression of the NOTCH3/GATAD2A signaling axis 57.

A group of circRNAs can inhibit the apoptosis of cancer cells. Xia at el. found that hsa_circ_0011385 suppresses the cell cycle arrest and apoptosis of PTC cells *in vitro* by targeting miR-361-3p via a miRNA sponge process 82. Similarly, Yang et al. demonstrated that hsa_circ_0039411 represses the apoptosis of PTC cells by functioning as a sponge for miR-1179 and miR-1205. Inhibition of miR-1179 and miR-1205 increases the expression levels of the mRNAs encoding ATP-binding cassette transporter A9 and metastasis-associated 1, respectively, at the post-transcriptional level 83. Furthermore, circ_0025033 promotes PTC cell proliferation, migration, and invasion, and inhibits cell apoptosis by acting as a sponge for miR-1231 and miR-1304 84. In addition to circRNAs that act as miRNA sponges, circ_0067934 interacts with proteins directly to promote TC cell proliferation, migration, and invasion, and inhibit cell apoptosis by promoting the EMT and PI3K/AKT signaling pathways 85.

To date, only one circRNA, namely circ-ITCH, has been found to be down-regulated in TC 86. Functional tests showed that overexpression of circ-ITCH inhibits the proliferation and invasion of PTC cells, and promotes cell apoptosis by binding to miR-22-3p and inhibiting its ability to up-regulate the expression of CBL (Cbl proto-oncogene), an E3 ligase targeting nuclear β-catenin. The resulting increase in CBL expression inhibits activation of the Wnt/β-catenin pathway, thereby inhibiting the progression of PTC.

**Figure 2:**
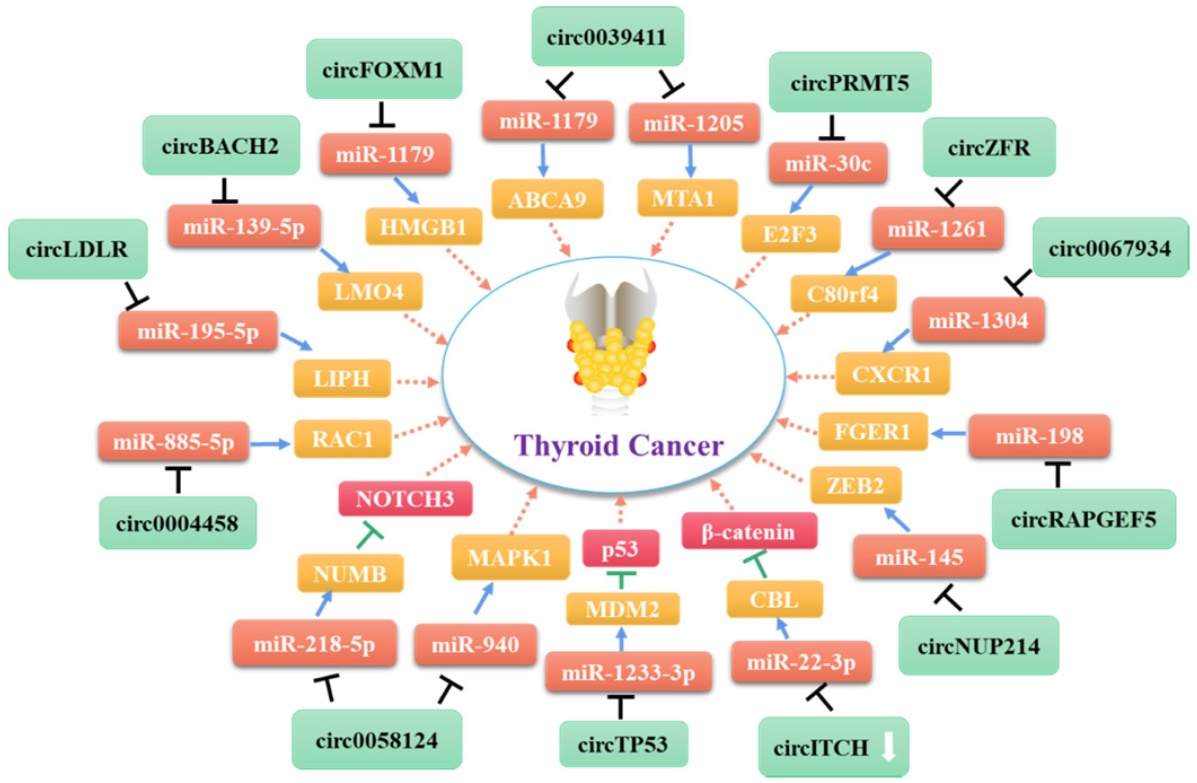
Molecular mechanism network of circRNA in thyroid cancer (CircRNAs serve as miRNA sponges).

**Figure 3:**
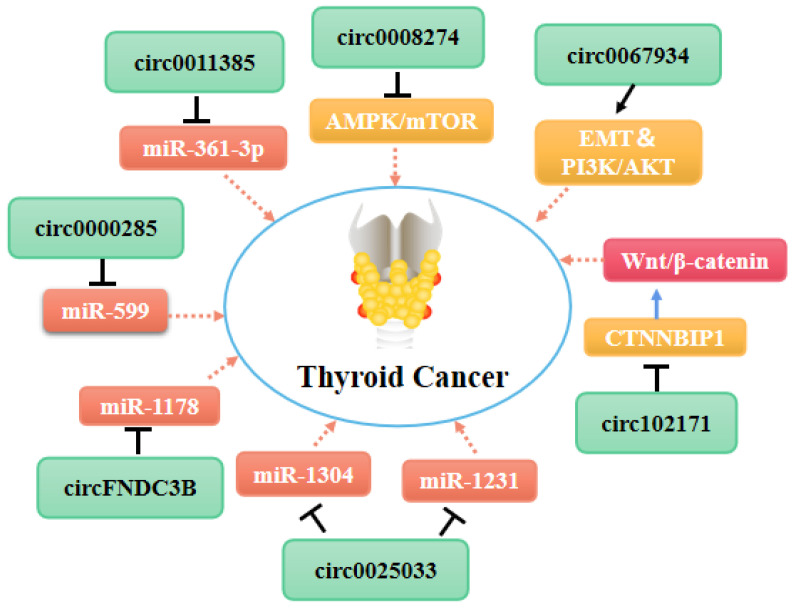
Molecular mechanism network of circRNA in thyroid cancer (CircRNAs bind with proteins or miRNAs).

As shown in **Table 1**, most of the circRNAs related to TC progression are up-regulated in tumor cells and exert their roles by functioning as miRNA sponges 75, 82, 86 (**Figure 2**). Some other circRNAs act directly on proteins or bind to miRNAs to control cancer progression (**Figure 3**). As mentioned above (see section 1.2.1), some circRNAs contain one or more miRNA binding sites 86 that enable them to sequester miRNAs and inhibit their ability to regulate the expression of their downstream target genes 87, 88. Indeed, circRNAs have been used widely as miRNA sponges to regulate target genes in cancer research. Using the GEO and TCGA databases, Liu et al. integrated circRNA, miRNA, and mRNA data from PTC tissues and non-tumor tissues to construct a circRNA-miRNA-mRNA regulatory network. This approach revealed that circRNAs regulate TC cell proliferation, apoptosis, and invasion by targeting miRNAs 88.

Overall, increasing efforts have been made to explore the mechanisms by which circRNAs regulate TC progression; such investigations have provided new insights into the pathogenesis of the disease.

### 2.3 CircRNAs as biomarkers for the diagnosis and treatment of TC

#### 2.3.1 CircRNAs as biomarkers for TC diagnosis

The incidence of TC, especially that of PTC, has increased rapidly over recent decades, and only 59% of patients with progressed TC have a 5-year overall survival rate 21. At present, diagnosis of thyroid tumors involves the use of ultrasound and radionuclide scanning, as well as various other procedures 89. The initial risk estimate, typically derived from ultrasound and sometimes cytology reports, should determine the need for treatment and the type, frequency, and length of subsequent follow-ups 90. However, accurate identification of TC from indeterminate cytological samples is challenging, and ultrasonographic features can be misleading in these types of tumors 91.

Traditionally, blood biomarkers have been widely used to diagnose cancer, but their low sensitivity and specificity has limited their applications for early diagnosis and prognosis 92. Therefore, biomarkers with high specificity and sensitivity are urgently needed. Recently, circRNAs were reported to be ideal liquid biopsy biomarkers. Additionally, circRNA expression patterns show significant differences between TC patients and healthy controls, and many circRNAs show tissue-specific and developmental stage-specific expression patterns and play critical roles in cancer-related biological processes 59. In view of their molecular stability, rich diversity, and tissue specificity, circRNAs in body fluids have potential as novel biomarkers for the monitoring of the development and progression of TC 93.

Shi et al. 94 found that the expression levels of two circRNAs (circRAPGEF5 and hsa_circ_0058124) were significantly higher in thyroid samples from PTC patients than in those from healthy controls or in benign thyroid nodules. In addition, the combination of the expression levels of these two circRNAs was independently correlated with the occurrence of PTC, indicating that they could be used as new diagnostic biomarkers of the disease. Other studies have shown that circMAN1A2 95, hsa_circ_0102272 96, hsa_circ_0124055 97, hsa_circ_0101622 97, hsa_circ_0137287 98, and circ_0006156 99 play important roles in regulating the proliferation, migration, and apoptosis of TC cells, and could also be used as biomarkers of PTC. Despite these findings, research into the potential use of circRNAs as diagnostic biomarkers of TC is still at an early stage. More detailed investigations are certainly required before circRNAs can be considered as biomarkers for clinical application. Nonetheless, the stability of circRNAs in exosomes and plasma suggests that they may represent a convenient method of diagnosing TC. In addition, the diversity and tissue-specific expression of circRNAs give them advantages over more traditional biomarkers, which tend to have low organ specificity.

#### 2.3.2 CircRNAs as therapeutic targets for TC

TC is a malignant tumor with an increasing incidence 17, 18. However, the current clinical approach to treatment of TC, which involves surgery and/or radiotherapy or chemotherapy, results in low recovery rates and high recurrence rates; hence it is not sufficiently effective 100. Moreover, radiotherapy can produce obvious side effects, while surgery can affect the normal physiological structure and function of patients 101, 102. Therefore, there is an urgent need to develop a new treatment strategy for thyroid tumors.

Targeted regulation of specific circRNAs may be a new therapeutic approach for thyroid tumor patients. Indeed, a number of studies have shown that various circRNAs, including circFOXM1 77, hsa_circ_0058124 57, 81, and hsa_circ_0039411 83, play a role in the growth, migration, invasion, and development of thyroid tumors. Recently, Zhou et al. found that high has-circ-0008274 expression increases PTC cell proliferation and invasion 52, and the has-circ-0008274/AMPK/mTOR axis may be a novel therapeutic candidate target for PTC. Other circRNAs, including circ-0004458 62, circ-BACH2 63, circ-FNDC3B 64, circ-0000285 103, play unique roles in regulating TC progression. These circRNAs could represent new therapeutic targets for thyroid tumors.

Drug resistance is a prominent problem associated with the treatment of thyroid tumors, especially anaplastic thyroid carcinoma (ATC) 104. Due to the undifferentiated phenotype and aggressive nature of ATC, resistance to conventional treatments such as radiotherapy and chemotherapy, including resistance to cisplatin, is observed often 105, 106. Therefore, patients with ATC tend to have a very poor prognosis. CircEIF6 can promote cisplatin-induced autophagy by regulating the hsa_miR-144-3p/TGF-α axis, thereby inhibiting cell apoptosis and enhancing the resistance of PTC and ATC cells to cisplatin 104. Therefore, it may be possible to enhance the sensitivity of thyroid tumor patients to cisplatin and to enhance the effect of chemotherapy by knocking out circEIF6.

To date, only a few studies have investigated the use of circRNAs as therapeutic targets for thyroid tumors, and further research is clearly needed.

## 3. CircRNAs and AITD

Although current understanding of the pathogenesis of HT remains very limited, it does depend on a number of factors, including genetic susceptibility and environmental and immune factors 107-110. Some investigations have shown that circRNAs play a role in the occurrence and development of HT. For instance, the expression level of hsa_circ_0089172 is increased markedly in HT patients and is inversely correlated with reduced levels of miR-125a-3p. Thus, hsa_circ_0089172 could be regarded as a potential diagnostic biomarker of HT and may play a crucial role in the pathogenesis of HT by acting as a miRNA sponge toward miR-125a-3p 111. However, research in this area is still in its infancy and only a few relevant studies have been published. Generating a complete profile of circRNA expression in HT patients would aid the identification of new biomarkers and the development of novel strategies for HT diagnosis and treatment.

CircRNAs may also play a role in the development of GD, another AITD. Sun et al. isolated serum exosomes from five primary GD patients and five healthy controls, and confirmed that hsa_circRNA_000102 was up-regulated significantly in those from GD patients 112. Moreover, analysis of the circRNA/miRNA/mRNA interaction network revealed the potential miRNA targets of hsa_circRNA_000102 and their related downstream target genes 112. This novel investigation provides a feasible framework for the identification and validation of circRNA biomarkers.

Thyroid-associated ophthalmopathy (TAO) is one of the most common clinical manifestations of GD patients; however, the mechanism underlying TAO is not fully understood. Wu et al. used high-throughput RNA sequencing of orbital fat/connective tissue samples to identify mRNAs and circRNAs that were differentially expressed between the control group and patients with TAO 113. In addition, Wu et al. revealed the roles of the differentially expressed circRNAs in TAO by circRNA-mRNA co-expression and circRNA/miRNA interaction analyses. The results of these analyses suggested that differentially expressed circRNAs may be involved in the pathogenesis of TAO, and that the circRNA_14940-CCND1-Wnt signaling pathway may be an important regulatory axis 113.

Overall, only a few studies have examined the role of circRNAs in the development of AITD, and research in the field is still in its infancy. The functions of the majority of circRNAs remain unclear, and their relationship to the occurrence and development of AITD require further investigation and clarification. The identification of circRNAs that are differentially expressed in AITD may provide insights into the pathogenesis of the disease.

## 4. Conclusions and perspective

TC and AITD are common thyroid diseases with high incidences. In recent years, the roles of circRNAs in diseases, including various cancers, have been investigated vigorously. In this review, we have systematically discussed the roles of circRNAs in TC and AITD, and described their potential use for the diagnosis and treatment of these diseases.

Accumulating evidence suggests that control of the transcriptome plays an important role in tumorigenesis and the progression of TC. Notably, circRNAs play a critical role in transcriptional and post-transcriptional regulation during tumorigenesis and TC development. As a relatively new focus of transcriptomic regulation, the molecular stability, substantial diversity, and tissue specificity of circRNAs have led to investigations of their use as diagnostic biomarkers of disease. Indeed, studies of circRNAs have deepened our understanding of the complex regulated network of non-coding RNAs. One of the main advantages of circRNAs as biomarkers is their high circulating stability and ability to be detected via RT-PCR analyses of minimally invasive blood, urine, or saliva samples. In addition, circRNAs might be useful therapeutic agents and/or targets. The use of circularized miRNA sponges in cells is a new candidate for RNA-based cancer treatments. Furthermore, examining the differences between circRNA levels in tumors and normal tissues is a topic for future clinical research, although large clinical datasets will need to be examined. However, at present, most research is focused on the relationship between circRNA levels and PTC, while other subtypes of TC are largely ignored. Although these subtypes do not account for most TC, they still have clinical research value.

In addition to TC, circRNAs play an important role in the pathogenesis of AITD, although research into this area is still in its infancy. A number of circRNAs are differentially expressed in various AITDs, but the mechanisms of action of these circRNAs are still unclear and require further verification. Examining the abnormal expression patterns of circRNAs during the development of AITDs will improve our understanding of the molecular basis of these disorders, and will provide a theoretical basis for the use of circRNAs as diagnostic markers and possible therapeutic targets of AITD.

Although many studies have shown that AITD is closely related to TC, the interaction and association in circRNA has not been studied. Future studies will be necessary to understand the connections between thyroid autoimmunity and cancer, to allow the design of tailored therapies for TC patients with AITD.

The potential use of circRNAs for clinical applications faces a number of challenges. First, the molecular mechanisms underlying the physiological and pathological effects of circRNAs remain largely unknown. Before they can be considered for use as biomarkers or therapeutic targets of TC and AITD, additional research is required to define their precise functions and mechanisms of action. Second, the naming standards for circRNAs are not uniform. Thus, numerous authoritative studies cannot be unified and generalized easily. Third, the potential use of circRNAs as therapeutic targets in clinical practice is currently at the stage of inference and theory; potential problems related to side effects, drug resistance, therapeutic effects, and administration mode remain unknown.

In conclusion, through the development of techniques to identify and screen novel circRNAs, as well as the improvement of online databases, it is possible that circRNAs will one day be used widely for the diagnosis, monitoring, and treatment of thyroid diseases.

## Figures and Tables

**Table 1:** Dysregulated circRNAs in thyroid cancer (TC)

Name	Dysregulation	Sponge target	Downstream target	Function	Material Source	Reference
circFOXM1	up-regulated	miR-1179	HMGB1	promotes PTC development	78 PTC and paired non-tumorous tissue samples	77
circ_0058124	up-regulated	miR-218-5p	NOTCH3/GATAD2A	promotes PTC proliferation, invasion, and metastasis and associates with the malignant features and poor outcomes	92 PTC and paired non-tumorous tissue samples	56
		miR-940	MAPK1		51 TC and paired non-tumorous tissue samples	81
circ_0039411	up-regulated	miR-1179	ABCA9	promotes PTC growth, migration, and invasion and suppresses cell apoptosis	46 patients with PTC	83
		miR-1205	MTA1			
circPRMT5	up-regulated	miR-30c	E2F3	promotes advanced stage and lymph node metastasis	55 PTC and paired non-tumorous tissue samples	80
circ_0011385	up-regulated	miR-361-3p	——	promotes TC proliferation, migration and invasion and suppresses cell cycle arrest and apoptosis	15 TC and paired non-tumorous tissue samples	82
circZFR	up-regulated	miR-1261	C8orf4	promotes TC proliferation, migration and invasion	41 PTC and paired non-tumorous tissue samples	75
circ-ITCH	down-regulated	miR-22-3p	CBL/Wnt/β-catenin	suppresses PTC proliferation and invasion and enhances apoptosis	37 PTC and 14 matched non-tumor tissues (all PTC patients did not receive any treatment before surgical removal of the tumor)	86
circ_0067934	up-regulated	miR-1304	CXCR1	suppresses TC apoptosis and promotes proliferation, migration, and invasion	50 TC and paired non-tumorous tissue samples (all TC patients did not receive any treatment before surgical removal of the tumor)	85
circ_0025033	up-regulated	miR-1231	——	promotes PTC proliferation, migration and invasion and suppresses cell apoptosis	8 PTC and paired non-tumorous tissue samples (all PTC patients did not receive any treatment before surgical removal of the tumor)	84
		miR-1304				
circTP53	up-regulated	miR-1233-3p	MDM2/p53	promotes cancer cell proliferation	25 PTC and paired non-tumorous tissue samples (23 cases were differentiated thyroid carcinoma, and 2 cases were poorly differentiated tumors)	76
circRAPGEF5	up-regulated	miR-198	FGFR1	promotes PTC proliferation and migration, a potential biomarker and therapeutic target	30 PTC and paired non-tumorous tissue samples (all PTC patients did not receive any treatment before surgical removal of the tumor)	114
circNUP214	up-regulated	miR-145	ZEB2	promotes PTC proliferation, migration, and invasion	30 PTC and paired non-tumorous tissue samples	79
circ_0004458	up-regulated	miR-885-5p	RAC1	promotes PTC progression, a potential therapeutic target and biomarker	48 PTC and paired non-tumorous tissue samples	61
circLDLR	up-regulated	miR-195-5p	LIPH	promotes PTC proliferation, migration, and invasion	60 PTC and paired non-tumorous tissue samples	78
circBACH2	up-regulated	miR-139-5p	LMO4	a tumor biomarker of PTC	40 PTC and paired non-tumorous tissue samples	62
circ102171	up-regulated	CTNNBIP1/Wnt/β-catenin		promotes `PTC progression	47 PTC and paired non-tumorous tissue samples	69
circ0008274	up-regulated	AMPK/mTOR		promotes PTC proliferation and invasion, a novel therapeutic candidate target	Primary PTC tissues and paired non-tumorous tissue samples	51
circ0000285	up-regulated	miR-599	--	promotes TC metastatic abilities, a potential therapeutic target	TC and paired non-tumorous tissue samples (all PTC patients did not receive any treatment before surgical removal of the tumor)	103
circFNDC3B	up-regulated	miR-1178	TLR4	a novel biomarker	PTC and paired non-tumorous tissue samples (all PTC patients did not receive any treatment before surgical removal of the tumor). For exosome purification, serum samples were collected from these 42 cases of PTC and 40 cases of healthy donors.	63

## Abbreviations

AITD: autoimmune thyroid disease
ATC: anaplastic thyroid carcinoma
CBL: Cbl proto-oncogene
ceRNA: competitive endogenous RNA
circRNA: circular RNA
FNA: fine-needle aspiration
GD: Graves' disease
HT: Hashimoto's thyroiditis
miRNA: microRNA
PTC: papillary thyroid carcinoma
p21: cyclin-dependent kinase inhibitor 1
TAO: thyroid-associated ophthalmopathy
TC: thyroid cancer
